# Analysis of 30 Patients with Acupuncture-Induced Primary Inoculation Tuberculosis

**DOI:** 10.1371/journal.pone.0100377

**Published:** 2014-06-24

**Authors:** Yangbo Liu, Jingye Pan, Keke Jin, Cailong Liu, Jing Wang, Li Chen, Lei Chen, Jiandong Yuan

**Affiliations:** 1 Orthopedics, The First Affiliated Hospital of Wenzhou Medical University, Wenzhou, Zhejiang, China; 2 Intensive Care Unit, The First Affiliated Hospital of Wenzhou Medical University, Wenzhou, Zhejiang, China; 3 Pathophysiology, Wenzhou Medical University, Wenzhou, Zhejiang, China; 4 Biomedicine, The University of Melbourne, Melbourne, Victoria, Australia; Fundació Institut d'Investigació en Ciències de la Salut Germans Trias i Pujol. Universitat Autònoma de Barcelona. CIBERES, Spain

## Abstract

Primary inoculation tuberculosis is a skin condition that develops at the site of inoculation of *Mycobacterium tuberculosis* in tuberculosis-free individuals. This report describes the diagnosis, treatment and >1 year follow-up of 30 patients presenting with acupuncture-induced primary inoculation tuberculosis. Our data provide a deeper insight into this rare route of infection of tuberculosis. We also review effective treatment options.

## Introduction

Tuberculosis (TB) has existed in humans for millennia [1]. It is estimated that approximately one third of the world's population is infected with *Mycobacterium tuberculosis* which causes 8.8 million new cases of tuberculosis accounting for approximately 1.1 million deaths each year [2].

Pulmonary tuberculosis is by far the most abundant form: extra-pulmonary tuberculosis accounts for about 20% of cases and tuberculosis of the skeleton-muscular system accounts for 10% of all infections. Interestingly, a low incidence (0.1%) of primary inoculation tuberculosis has also been reported [3]–[5].

In the United States, extra-pulmonary tuberculosis accounts for 18% of all tuberculosis infection, with cutaneous tuberculosis representing 1.8% of cases [6]. Few case reports of primary inoculation tuberculosis are available, with only 33 cases being described between 1935 to 2012. The largest number of cases described in the literature is five [7].

Given the difference in invasive routes between primary inoculation and pulmonary tuberculosis, no generalization can be made as for incubation period, clinical features, treatment and prognosis of primary inoculation tuberculosis based on lung disease case reports to guide clinical practices. In this study, we described 30 cases with primary inoculation tuberculosis (7 confirmed and 23 suspected) that developed over a short period of time. All subjects were followed up for at least one year to assess the effectiveness of a combination of drug and surgical intervention in treating primary inoculation tuberculosis.

## Case Description and Methods

### Patients and source of infection

Seven confirmed and 23 suspected, total 30 patients (13 male and 17 female) with primary inoculation tuberculosis were selected from the same clinic in Wenzhou City, China that specialized in treatment of muscle and soft tissue pain and osteoarthritis of the knee. Seven confirmed cases ages ranged from 31 to 67 years (mean: 53.14 years), and total 30 patients ages ranged from 31 to 71 years (mean: 52.3 years).

They had all undergone acupuncture and electrotherapy, administered by the same clinician, once every two days for about two weeks for the treatment of neck, back, elbow, wrist, hip, knee and ankle pain. The procedures took place between May 2011 and August 2011. All the patients were screened by chest X-rays for pulmonary tuberculosis. Only one suspected patient had a history of inactive pulmonary tuberculosis lesions on chest X-rays with negative sputum examination.

Three suspected patients had a medical history of type 2 diabetes. All subjects were negative for HIV.

In most patients the electrotherapy lasted 10–30 min. All injection materials were disposable. A total of four electrotherapeutic pads were used, without disinfection.

Most patients linked the lesion sites to electrotherapy. Therefore, it was speculated that the occurrence of tuberculosis infection might have resulted from the introduction of tubercle bacilli from the electrotherapeutic pads, into soft tissues via small skin wounds. However, culture of samples from the four electrotherapeutic pads appeared negative. Accordingly, it was impossible to determine the source of contamination.

Over the same time period, a total of 58 subjects had undergone invasive treatment at the clinic. There were three cases with unknown condition due to loss of contact, and 25 cases that did not contract infection. 18 patients out of the 55 received triamcinolone acetonide treatment at the acupuncture sites. The same batch of un-used drugs was tested to be qualified and effective. The treatment procedure with triamcinolone acetonide was triamcinolone acetonide + vitamin B12 once every other day for six months prior to the outbreak. On average, they had received 6–8 cycles of the treatment. Within the 18 patients, 12 were infected with TB, six were not. For the 37 of the 55 patients, who did not received triamcinolone acetonide treatment, 18 patients were infected with TB, 19 didn't; there was no significantly statistic difference between triamcinolone acetonide and non-triamcinolone acetonide treatment with chi-square test. The results showed that susceptibility of *Mycobacterium tuberculosis* infection in these patients is not affected by glucocorticoids.

The study was approved by the Ethics Committee of the First Affiliated Hospital of Wenzhou Medical Unversity. Written informed consent was obtained from every participant.

### Diagnosis

Purified protein derivative (PPD) skin test was performed in all 30 patients. The PPD tests of all patients were positive.

Quantitative fluorescent PCR assay, rapid identification of *Mycobacterium tuberculosis* Beijing strains, Rifampicin (RFP) susceptibility testing of *M. tuberculosis*, detection of isonicotinylhydrazine (INH)-resistant gene mutation, and variable number tandem repeat (VNTR) genotyping were performed on samples from six patients using Tuberculosis Drug Resistance Detection Array Kit (CapitalBio) and Mycobacteria Identification Array Kit (CapitalBio). These six patients did not receive any anti-tuberculosis drugs before the PCR assy. The results showed four isolates were caused by RFP and INH sensitive *M. tuberculosis* Beijing strains, and VNTR genotyping revealed that the isolates were of the same genotype, and the other two isolates were negative.

Other eight cases in all 30 patients, biopsied, lesion smears and tubercle bacillus culture from wound saline solution were performed using BacT/ALER MP culture media (BIOMERIEUX). Acid-fast bacillus was found in all the smears, and positive tubercle bacillus culture was found in three patients (positive rate: 37.5%). The three positive samples were sensitive to INH and RFP in susceptibility testing, no test for other drugs were performed. Prior to the biopsy and culture, these eight patients all had receive a 1-week's quadruple anti-tuberculosis drugs treatment (RFP/INH/PZA/EMB). The results of these eight patients' routine bacterial culture were negative.

In general, there were seven confirmed patients in this cutaneous tuberculosis outbreak while the rest twenty-three were supposed to be suspected patients.

**Figure 1:**
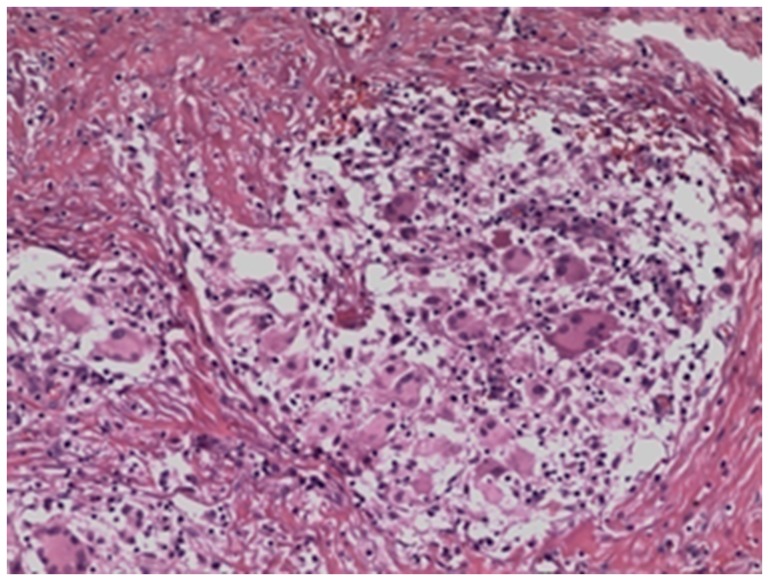
Infiltration of a large number of chronic inflammatory cells among muscle fibers. Many giant cells and epithelioid cells aggregated and formed multiple tubercles. Microabscess was present in the lesion region, and granulomatous inflammatory alteration was detected.

The pathologic examination results are shown in Figure 1.

### Clinical manifestations of seven confirmed patients

**Figure 2:**
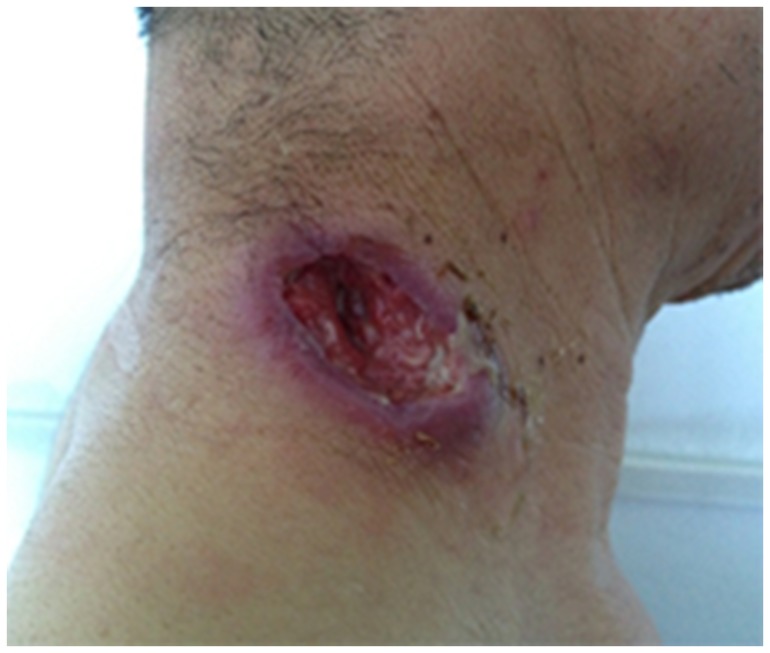
A fresh lesion on the right side of the neck. The ulcer was oval, with the largest diameter of about 5

The incubation period defined as the time between the first invasive treatment and onset of clinical symptoms (subcutaneous mass, local redness, swelling and pain, and fever) ranged from 1.5 to 4 weeks (mean, 2.5 weeks). The lesions were widely distributed at sites of puncture. Figure 2 shows a representative photograph of a typical lesion.

**Figure 3:**
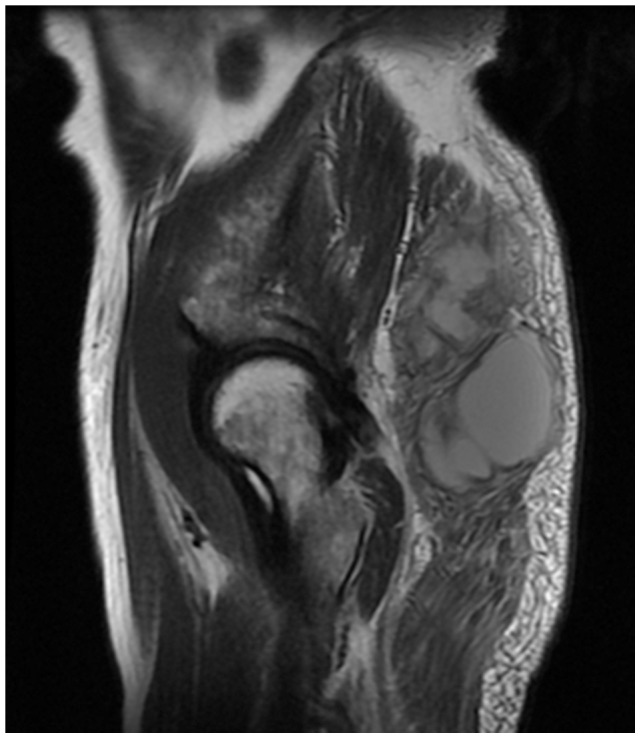
MRI scan of the hip on T1W1 reveals elevated signal intensity on the right gluteus maximus muscle. Massive irregular, homogeneous, mass-like abnormal signal intensity with unclear boundary was observed in the gluteus maximus muscle, measuring about 151 cm×90 cm, and subcutaneous edema-like signal intensities were observed in the right hip.

Prior to receiving regular anti-tuberculosis drugs therapy, all seven confirmed cases had ulcerative lesions, which formed sinus tracts. The neighboring lesions developed ulcers or expanded to the neighboring soft tissues to form giant abscesses (Figure 3). Six confirmed patients developed symptoms of severe infection with high fever, including hyperpyrexia (axillary temperature >39.1°C) and shivering. One patient had symptoms commonly associated with tuberculosis, including low-grade fever, night sweats, anorexia and marasmus.

**Figure 4:**
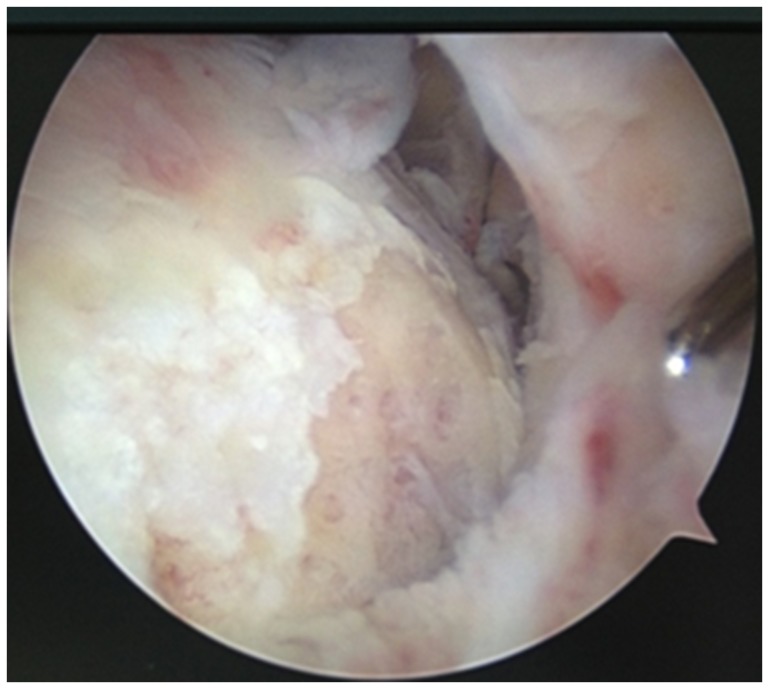
Hyperplasia of the knee joint synovium, grey and dark in color, with a soft texture, complicated by necrotic tissues. Some knee joint synovium underwent caseous changes, and articular cartilage necrosis and desquamation were observed. Subchondral bone was exposed, and vermiform bone destruction was observed on the condyle of femur and the margin of the tibial plateau.

One confirmed patient developed tuberculosis infection of the knee joint, characterized by swelling, positive patellar tap test, limited flexion and extension, ulceration and suppuration of the needle tract. Knee arthroscopy showed hyperplasia of the knee joint synovium, with grey and dark color and soft texture, complicated by necrotic tissues. In addition, knee joint synovium underwent caseous changes, and articular cartilage necrosis and desquamation was observed. Subchondral bone was exposed, and there was evidence of vermiform bone destruction detected on the condyle of femur and the margin of the tibial plateau (Figure 4). Positive joint fluid smear test for acid fast bacilli was detected.

**Figure 5:**
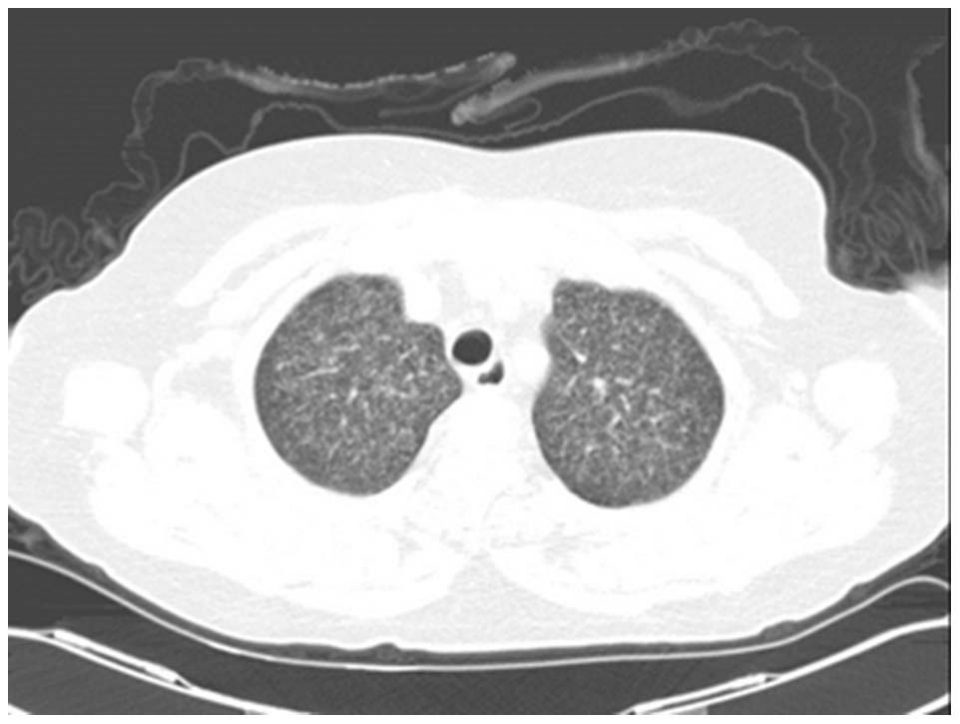
Left and right upper lungs displayed diffuse, ground-glass shadows with increased intensity.

**Figure 6:**
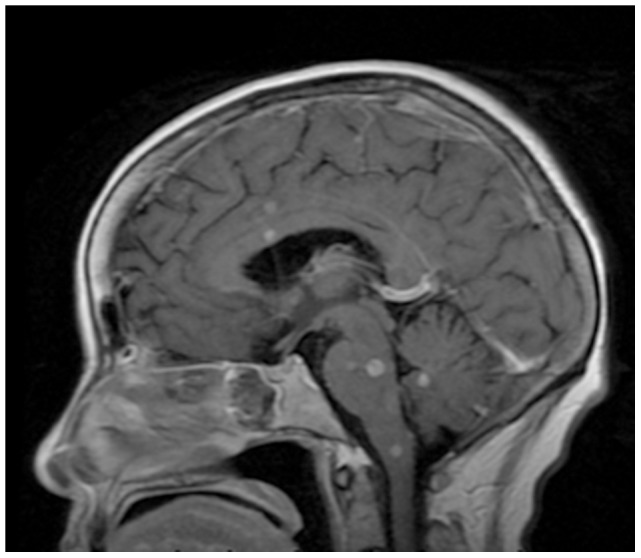
Enhanced MRI scan shows tuberculous, ring-shaped, signal-intensified lesions in bilateral frontal and parietal lobes, centrum semiovale, peri-lateral ventricular region, basal ganglia regions, thalamus, brainstem and cerebellum.

Two severe cases were found with tuberculous meningitis and miliary pulmonary tuberculosis complications (Figure 5), including one case admitted to an intensive care unit (ICU). Tuberculous meningitis was characterized by tuberculous lesions with low-intensity signals on T1-weighted imaging (T1WI), high-intensity signals around T2WI, slightly low-intensity signals on central zone of T2WI. In addition, there were slightly high-intensity signals on DWI of the bilateral frontal and parietal lobes, centrum semiovale, peri-lateral ventricular region, basal ganglia regions, thalamus, brainstem and cerebellum. Enhanced MRI scan showed the appearance of ring-shaped, signal-intensified lesions (Figure 6).

### Clinical manifestations of 23 suspected patients

The incubation period of 23 suspected patients ranged from 1 to 12 weeks (mean, 3.9 weeks). 18 cases had ulcerative lesions, which formed sinus tracts. Among the five patients without sinus tracts, two cases had cold abscesses with normal local skin temperature, and three had an elevated local skin temperature on the lesion together with inflammatory soft tissue reactions, characterized by redness and swelling. Ten patients had high fever, four patients had low-grade fever, night sweats, anorexia and marasmus. The nine remaining cases (30.0%) had local symptoms without systemic manifestations. One patient developed tuberculosis infection of the knee joint, and the clinical manifestation was similar to the confirmed one. One severe case from suspected patients was found with tuberculous meningitis and miliary pulmonary tuberculosis complication.

### Treatment options

Since the 30 patients were treated at the identical clinic, developing the similar clinical manifestations, using the close therapeutic program during the short period of time, suspicions were high that the suspected patients were infected by the same tuberculosis as the confirmed patients. Hence the analogous anti-tuberculosis drug treatment protocols were chosen for all the infected patient. Fortunately, the good treatment outcomes, in turn, proved that our speculation was very close to the actual situation.

All 30 patients had onset of primary inoculation tuberculosis within 1 to 3 months of the acupuncture procedure. Prior to definitive diagnosis at our hospital, 25 cases (including seven confirmed cases) had received various treatments at different hospitals or clinics, and the other five late onset cases had not been treated. Treatment strategies were classified into surgical treatment alone, treatment with anti-tuberculosis drugs independently, and combination treatment with surgery and anti-tuberculosis drugs.

The anti-tuberculosis drug treatment protocols were developed according to the published guidelines of the American Thoracic Society (2003 version) [8]. The duration of pyrazinamide (PZA) and ethambutol (EMB) treatment was extended in some patients according to the actual therapeutic efficacy and side effects. Other drugs were changed due to side effects or allergy. Therapeutic efficacy was retrospectively evaluated.

### Previous treatments

Five confirmed patients underwent surgical debridement procedures prior to definitive diagnosis of primary inoculation tuberculosis. The wound failed to heal after either primary suture or open dressing without regular administration of anti-tuberculosis drugs. One confirmed and three suspected patients with abscess formation as revealed by imaging, received anti-tuberculosis agents for one week followed by debridement to remove the abscesses. In these subjects, no ulceration was found after primary suture of the wound. One confirmed and eight suspected patients presenting with minor wounds or no ulcerative primary lesions received anti-tuberculosis drugs alone. This achieved gradual reduction and healing of the lesions.

13 suspected patients underwent between two and five surgical debridement procedures before they were told and concentrated to our hospital. Without regular administration of anti-tuberculosis drugs, the wound failed to heal after either primary suture or open dressing. In all cases, the wound healed slowly after the final debridement procedure combined with anti-tuberculosis drugs.

### Previous quinolone antibiotics efficacy of total 30 patients

Twenty-five patients underwent antibiotic therapy before anti-tuberculosis drugs administrations, including 17 who received using quinolone antibiotics. Fifteen of the 17 (88.2%) showed a remarkable alleviation of systemic symptoms and redness, as well as reduced swelling of the local lesions. There was also an extended interval before the reemergence of ulceration requiring debridement. No obvious efficacy was achieved in two of the 17 patients using quinolones.

Among eight patients treated with non-quinolone antibiotics, one had alleviation of systemic symptoms, and reduced redness and swelling of focal lesions (this patient received ceftriaxone). Treatment with non-quinolone agents was ineffective in the other seven cases. The 30 patients were divided into quinolone-treatment group and non-quinolone-treatment group. Numbers of relieved and non-relieved were recorded, and analyzed with the Fisher's exact test to compare the difference in relieving rates between the two groups (*P* = 0.001), the result revealed that quinolone antibiotics appeared more effective against tubercle bacillus than non-quinolone non-anti-tuberculosis agents.

## Follow Up and Treatment Results

All patients received anti-TB treatment with a combination of four drugs for 2–6 months, depending on the alleviation of systemic symptoms and wound healing. The sequential enhanced treatment with two anti-tuberculosis drugs was ranged from 7 to 12 months. Nine suspected patients with good responses to drug therapies were in group I, their systemic symptoms were completely resolved (or they did not have any symptoms at all from the beginning) and their skin lesions were healed after a 2-month's quadruple anti-tuberculosis drugs (RFP/INH/PZA/EMB), and then this therapy was replaced by two anti-tuberculosis drugs (RFP/INH) for seven months. Due to gout and gastrointestinal reactions, PZA was replaced by Avelox (moxifloxacin hydrochloride and sodium chloride injection) in one suspected patient. PZA was used in the other eight suspected patients in the quadruple anti-tuberculosis drugs therapy. Four confirmed and ten suspected patients with moderate responses to drug therapies were in group II. Their skin lesions were healed between two and four months after the quadruple anti-tuberculosis treatment (RFP/INH/PZA/EMB) started, and then the therapy was replaced by two anti-tuberculosis drugs (RFP/INH) for 8–9 months. In four confirmed and eight suspected patients, the quadruple anti-tuberculosis drugs (RFP/INH/PZA/EMB) were administrated for four months. And two anti-tuberculosis drugs (RFP/INH) were adopted for sequential 8-months. Two suspected patients received a combination of rifapentine, INH, PZA and EMB due to allergy to RFP. Therapy continued with rifapentine-INH combination therapy for nine months.

Seven (3 confirmed and 4 suspected) patients with poor responses to drug therapies were in group III. Their skin lesions were healed between four and six months after the quadruple anti-tuberculosis drugs (RFP/INH/PZA/EMB) were treated, and then this therapy was replaced by two anti-tuberculosis drugs (RFP/INH) for 12 months. Due to liver dysfunction, PZA was stopped in one suspected patient at the 4th month and the three drugs (RFP/INH/EMB) were continued for another two months. PZA was used in the other six patients in the quadruple anti-tuberculosis drugs therapy (RFP/INH/PZA/EMB) for six months. The sequential 12-month's dual anti-tuberculosis drugs therapy (RFP/INH) was adopted in all of the seven patients.

Table 1 specifically lists the information of items of age, gender, diagnostic method, incubation period, sinus, high fever, severe case, operation, anti-tuberculosis drug treatment protocols and efficacy. We found no statistical significance in drug efficacy between the sinus/non-sinus, high fever/non-high fever or operation/non-operation groups. However, significant difference was observed between severe/non-severe patients in drug efficacy.

**Table 1:** Information on clinical features and treatments in 30 patients.

Patient No.	Age/Gender	Diagnostic method	Incubation period (weeks)	Sinus	High fever	Operation	Anti-tuberculosis drug treatment protocols (months)	Efficacy of anti-TB drugs(group)	Follow-up time
	Confirmed patients								
01	67,M	PPD+, PCR+	3	+	+	+	4(RFP/INH/PZA/EMB) & 8(RFP/INH)	II	36
02	54,F	PPD+, PCR+	2	+	+	+	6(RFP/INH/PZA/EMB) & 12(RFP/INH)	III	28
03	53,M	PPD+, PCR+	2	+	+	+	4(RFP/INH/PZA/EMB) & 8(RFP/INH)	II	40
04^K^	50,M	PPD+, smear+, C+	1.5	+	+	+	4(RFP/INH/PZA/EMB) & 8(RFP/INH)	II	33
05*	50,M	PPD+, smear+, C+	2	+	+	+	6(RFP/INH/PZA/EMB) & 12(RFP/INH)	III	38
06*	67,F	PPD+, smear+, C+	3	+	+	+	6(RFP/INH/PZA/EMB) & 12(RFP/INH)	III	38
07	31,F	PPD+, PCR+	4	+	-S	-	4(RFP/INH/PZA/EMB) & 8(RFP/INH)	II	30
	Suspected patients								
01^D^	71,F	PPD+	2	+	+	+	6(RFP/INH/PZA/EMB) & 12(RFP/INH)	III	32
02	45,F	PPD+	3	+	-	+	2(RFP/INH/PZA/EMB) & 7(RFP/INH)	I	36
03	40,M	PPD+	4	+	-	+	4(RFP/INH/PZA/EMB) & 8(RFP/INH)	II	30
04	60,F	PPD+, PCR-	3	-	-	-	4(RFP/INH/PZA/EMB) & 8(RFP/INH)	II	38
05	64,M	PPD+	6	-	-	-	4(RFP/INH/PZA/EMB) & 8(RFP/INH)	II	36
06	60,F	PPD+	4.5	+	+	-	2(RFP/INH/PZA/EMB) & 7(RFP/INH)	I	40
07	51,M	PPD+	3	+	+	-	2(RFP/INH/PZA/EMB) & 7(RFP/INH)	I	28
08^D^	56,M	PPD+	5	+	-	-	2(RFP/INH/PZA/EMB) & 7(RFP/INH)	I	35
09	54,F	PPD+	3	+	-	+	2(RFP/INH/PZA/EMB) & 7(RFP/INH)	I	33
10	48,F	PPD+	4	+	-	+	2(RFP/INH/PZA/EMB) & 7(RFP/INH)	I	41
11^D^	59,F	PPD+	4	-	-S	+	4(RFP/INH/PZA/EMB) & 8(RFP/INH)	II	29
12	50,M	PPD+, smear+, C-	2	+	+	-	2(RFP/INH/Avelox/EMB) & 7(RFP/INH)	I	39
13	45,F	PPD+, smear+, C-	1.5	+	-S	-	6(RFP/INH/PZA/EMB) & 12(RFP/INH)	III	36
14	48,F	PPD+	4	+	+	+	4(RFP/INH/PZA/EMB) & 8(RFP/INH)	II	35
15^K^	48,M	PPD+	3	-	+	+	2(RFP/INH/PZA/EMB) & 7(RFP/INH)	I	36
16*	54,F	PPD+	2	+	+	+	6(RFP/INH/PZA/EMB) & 12(RFP/INH)	III	35
17	44,F	PPD+	1	-	+	+	2(RFP/INH/PZA/EMB) & 7(RFP/INH)	I	34
18	40,M	PPD+, smear+, C-	12	+	-	+	4(RFP/INH/PZA/EMB) & 8(RFP/INH)	II	28
19	50,M	PPD+, smear+, C-	2	+	-S	+	4(RFP/INH/PZA/EMB) & 8(RFP/INH)	II	29
20	36,F	PPD+, PCR-	4	+	+	+	4(Rifapentine/INH/PZA/EMB) & 9(Rifapentine/INH)	II	40
21	60,F	PPD+, smear+, C-	2	+	+	+	4(Rifapentine/INH/PZA/EMB) & 9(Rifapentine/INH)	II	38
22	55,F	PPD+	6	+	-S	+	4(RFP/INH/PZA/EMB) & 2(RFP/INH/EMB) & 12(RFP/INH)	III	32
23	60,M	PPD+	8	+	-	+	4(RFP/INH/PZA/EMB) & 8(RFP/INH)	II	27

+ : positive;

- : negative;

*: severe case,

K : knee TB,

D : type 2 diabetes,

S : symptoms commonly associated with tuberculosis, including low-grade fever, night sweats, anorexia and marasmus.

Good efficacy of drug treatment in nine suspected patients named group I; moderate efficacy of drug treatment in four confirmed and ten suspected patients named group II; poor efficacy of drug treatment in three confirmed and four suspected patients named group III.

Group I had one suspected patient and group II had one confirmed patient with tuberculosis of the knee joint. Arthroscopic knee debridement was performed after a 4-week's quadruple anti-tuberculosis drugs therapy (RFP/INH/PZA/EMB) and continuous lavage and drainage using streptomycin saline solution (1 g in 500 ml) was given for one week. Wound of the suspected patient in group I was healed in five weeks. The skin wound was completely healed in the 17th week of the quadruple anti-tuberculosis drugs therapy in the confirmed patient in group II. In the final follow-up, the knee functions of both patients were normal and without obvious sequelae. Two confirmed and one suspected cases in group III complicated by intracranial and pulmonary tuberculosis were treated with four anti-tuberculosis drugs. After four months of treatment, cerebral spinal fluid test was negative, and the chest CT scan displayed no tuberculous lesions. The patients continued treatment with four anti-tuberculosis drugs for two months and two drugs for 12 months (total of 18 months).

Seven confirmed patients and the seven confirmatory tests negative ones were compared. No significantly statistics difference was found in the incidence of sinus (*P* = 1.000) and high fever (*P* = 0.559) with Fisher's exact test. Incubation period and efficacy of anti-tuberculosis drugs also had no statistical difference between the confirmatory tests positive patients and negative ones with chi-square test (*P* = 0.134) and Student's t test (*P* = 0.121) respectively.

In cases where liver injury occurred, treatment was interrupted for three weeks during which liver-protective therapy was administered, before resuming with the anti-tuberculosis drug regimen.

After a mean follow-up of 34.33 weeks (range: 27–42 weeks), all lesions were healed. Two suspected patients still presented with subcutaneous nodules, but without newly formed lesions. The three cases with intracranial and pulmonary tuberculosis complications were negative for cerebral spinal fluid test as well as chest CT scan. No cases of recurrent tuberculosis were detected.

## Discussion

Again, on account of the similar epidemiological history, clinical manifestation and treatment effects, along with the four coincident VNTR genotyping results, the authors still considered the 30 cases as one outbreak of infection caused by the identical pathogen and discuss them together.

Primary inoculation tuberculosis is an infection of *Mycobacterium tuberculosis*, which usually results from direct introduction of the bacterium into the skin of a tuberculosis-free person. In most cases, minor injuries are found on skin [9]. The typical histological features of primary inoculation tuberculosis involve granulomatous nodules with infiltration of epithelioid cells, Langerhans' cells and mononuclear macrophages. However, these characteristics are not specific to tuberculosis, and other infectious lesions, e.g. from mycoses may present similar manifestations.

Since Laennec's description of the “prosector's wart” in 1826, science has made great strides forward. The cutaneous forms of the infection with *Mycobacterium tuberculosis* are various [10]. Such cutaneous infection is general via single superficial skin hurt, time and frequency of the contact with *Mycobacterium tuberculosis* are very short. The use of Chinese acupuncture needles which are able to deeply penetrate into the tissues surrounding tendons and nerves provide an ideal route for the inoculation of tuberculosis. The patients in our outbreak underwent acupuncture twice daily for two weeks. This high degree of potential exposure may explain why there were no cases of spontaneous healing. It was also possible that patients with self-healing of skin lesions had ignored their short-lasting symptoms and became the so called ‘non-incident’ patients.

PPD results of 30 patients were positive, indicating that PPD test was sensitive to primary inoculation tuberculosis. Due to factors of funding and patient compliance, we were unable to perform diagnosing experiments of biopsy, culture and PCR for each patient. We thought PPD trial could serve as a key diagnosing, since these patients were treated at the same clinic, with the parallel therapeutic program and during the short period of time. However, PPD is only a screening measure for sporadic cases. Nevertheless, if it were combined with clinical symptoms, its diagnostic reliability would greatly increase, particularly when the diagnosed specimens are hard to obtain. Goette, D. K., et al. thought a transition from a negative result to positive one in PPD trial could serve as a diagnostic criterion for primary inoculation tuberculosis [3], but it is difficult to get a result of PPD trial in the real clinical atmosphere before onset.

Previous literatures have reported that quantitative fluorescent PCR assay has a high sensitivity and specificity, but there continues to be no more than 25% of the false-negative rate [11]–[13]. Improper operation can cause *Mycobacterium tuberculosis* lost and specimen contamination. On the other hand, no tissue fluid around the lesions will also reduce the detection rate, and affect the authenticity of the results. Of course, we cannot ignore the possibility that there was no *Mycobacterium tuberculosis* exist in those samples at all. Negative tubercle bacillus culture rate was up to 62.5%. The most likely cause may be the effects of the anti-tuberculosis drug treatment which low the bacterial activity. Other possibilities include L-form conversion of *Mycobacterium tuberculosis* and technical error caused by improper operation. There was no statistical difference in incubation period, incidence of sinus, incidence of high fever and efficacy of anti-tuberculosis drugs between the patients of positive confirmatory tests and their negative counterparts. Therefore, we believe that the possibility of false-negatives is very big.

Only one patient (in suspected group) in our study had a medical history of tuberculosis. This patient was not admitted to the acupuncture clinic earlier than the other cases. In addition, three sputum smear examinations were all negative, and the pulmonary lesions did not indicate active tuberculosis. Therefore, the case was not considered as the source of infection. Despite the unsuccessful identification of the source of contamination, it is apparent that these infections were linked to acupuncture and moxibustion, because the 30 patients had the same epidemiological characteristics. Several previous studies have demonstrated that tuberculosis can occur as a result of tattoo or acupuncture treatments [14]–[16].

Most of the 30 patients had multiple skin infections, but the lesions were located to the sites of acupuncture and electrotherapy. Lesion severity and drug reactions in individual patient were similar, but we did not know whether these multiple lesions were independent or the result of the inoculation infections in the wounds via hemo-disseminated *Mycobacterium tuberculosis*. Although, occurrence of the three patients with meningeal and pulmonary tuberculosis and two patients with knee tuberculosis had confirmed the hemo-disseminated ability of this primary inoculation *Mycobacterium tuberculosis* to other tissues and the compartments.

To account for possible deviations in patients remembering the exact dates, the incubation period was recorded in weeks instead of days. In this study, the incubation period ranged from 1 to 12 weeks, in good accordance with similar studies [14], [15].

The lesions in our patients were distributed throughout the body at sites of puncture, indicating that inoculation of tubercle bacilli has no obvious preference for soft tissues. Indeed, tuberculosis can occur at neck (abundant blood supply) and muscle-tendon tissues (relatively less blood supply) with different characteristics compared with knee-joint tuberculosis that predominantly occurs at weight-bearing sites.

Almost all patients had subcutaneous nodules that were not completely inhibited by routine antibiotics including quinolone agents, and the resulting lesions did not readily heal. The lesions sizes ranged in size from a needle tip diameter to a diameter of 8 cm. They were characterized by the presence of a thick basement, and granular protrusions with bleeding upon touch. Ulcers surrounded by scarring and pigmentation were very slow to heal. Examination of the lesion smears showed positive acid-fast stained bacillus. Most lesions were relatively independent, although one patient presented with large cold abscesses on hip and waist. These findings indicate that primary inoculation tuberculosis lesions occur relatively independently, seldom with a local distribution.

There were three cases of tuberculous meningitis and military pulmonary tuberculosis complications. Our data suggest that 10% primary inoculation tuberculosis may develop hematogenous dissemination, which is rarely described in previous case reports. The occurrence of knee-joint tuberculosis demonstrated that invasive tubercle bacilli can be inoculated into a limited space, and we showed that satisfactory knee-joint function could be achieved through arthroscopic synovial debridement and lavage with streptomycin.

It is well recognized that debridement without anti-tuberculosis agents does not control tuberculosis, and that multiple debridements may cause deeper and wider local infections. In our series, surgical debridement was undertaken 2–4 weeks after administration of anti-tuberculosis agents to remove abscesses. Local lesions were lavaged with streptomycin, and the wound could heal with primary suture. Following surgery, more effusion fluid was released than in a normal wound. However, the wound was closed after at least two weeks. Two patients presented subcutaneous nodules after non-surgical drug therapy, no recurrence was observed after follow-up completion, and these patients rejected surgical removal of the nodules. But, the final outcomes required longer follow-up periods. Lesions without abscess were all healed following anti-tuberculosis drugs therapy. Therefore, treatment with anti-tuberculosis is sufficient to achieve satisfactory therapeutic efficacy, while the option of surgical treatment depends on abscess formation.

Our treatment plans with a combination of four or two anti-tuberculosis drugs for 6–9 or 9–12 months are recommended for treatment of tuberculosis. This is in accordance with the guidelines for treatment of tuberculosis as proposed by the American Thoracic Society [8]. To optimize the treatment regimen, patients were followed up every month, and the treatment regimen was adjusted as clinically indicated. Treatment with a four drug combination for two months, followed by a two drug combination for seven months (total of nine months) provided a satisfactory response for small primary lesions. However, treatment with a four drug regimen should be extended to six months, with total treatment duration of 18 months for patients without obvious improvement in wounds after four months of therapy, and for cases with intracranial and pulmonary tuberculosis complications.

Due to safety and ethical considerations, it was impossible to compare the relative therapeutic efficacy of treatments with various drugs at different cycles. Some patients stopped drug intake due to side effects. Nevertheless, all patients were treated for at least nine months, and all wounds were healed with no resistant strains detected. These findings show that drug sensitivity testing in combination with regular anti-tuberculosis drugs therapy achieves efficacy in the treatment of primary inoculation tuberculosis without recurrence.

Drug resistance is the predominant factor affecting efficacy but this was not seen in this outbreak. The bacteria counts and immune state are also important factors affecting symptoms and efficacy [10].

Based on the description of symptoms by patients, we simply divide the local symptoms into sinus and non-sinus groups and the systemic symptoms into high fever and non-high fever groups (since low fever and normal state were hard to recall and identify. High fever was easier to identify, and was, therefore, chosen as the parameter). The presence of sinus and high fever did not affect drug efficacy or antimicrobial sensitivity to the drug. Surgical treatment did not influence the sensitivity of the drug. In fact, efficacy was inferior in patients with severe conditions than in those with milder lesions.

There were only three severe patients who had meningeal or pulmonary tuberculosis. The efficacy of drug therapy in these patients was undoubtedly poor, which might be the result of the large amount of *Mycobacterium tuberculosis* entering into the cerebrospinal fluid where drugs were hard to reach.

Interestingly, glucocorticoids has been used in combination with electroacupuncture for the treatment of tuberculosis [17], [18]. Based on cytological findings, Rook, G., et al. reported that glucocorticoids play an important role in the process of tuberculosis infection. They suggested that a local increase in glucocorticoids will raise susceptibility to tuberculosis [19], [20]. However, our findings, which were contrary to previous experimental results, show that no statistical significance was found, and glucocorticoids did not affect the susceptibility of primary inoculation tuberculosis. The causes may be that the earlier results were just confined to animal experiments and cytological studies. *In vitro* and animal experiments cannot completely simulate the real human *Mycobacterium tuberculosis* infection. Another reason may be that glucocorticoids do affect the incidence of primary inoculation tuberculosis, but because of small sample size of this study and the lost to follow-up for 3 patients we didn't get the positive result. Anyway, the risk of *Mycobacterium tuberculosis* infection should be given high priority when invasive procedures are involved, and glucocorticoids should not be used blindly, frequently nor excessively.

## Conclusions

Based on our findings we make the following recommendations:

*Mycobacterium* can easily spread without proper microbiological control of these procedures. To this end, it was recently suggested that herbal medicine and acupuncture professions should also develop a system of statutory regulation [21] which should help prevent these issues.

Diverse symptoms (from subcutaneous induration to systemic dissemination or even intracranial infection) may appear after *Mycobacterium tuberculosis* infection. Therefore, infection of *Mycobacterium tuberculosis* should be excluded in patients with a history of invasive treatment, repeated symptoms or poor antibiotic efficacy. This would avoid large-scale outbreaks, similar to the one studied here.

For lesions with abscesses, surgical removal should be adopted in combination with anti-tuberculosis drugs, since operative removal alone is ineffective and may even make the condition worse. Different drug reactions may appear even among patients infected by the same *Mycobacterium tuberculosis* spp.

Classical anti-tuberculosis drug treatment programs are effective against lesions of the primary inoculation tuberculosis and with continued treatment, all patients can be cured.
